# When Feeding Tubes Cause Biliary Disease: A Case of Acalculous Cholecystitis Due to Gastrostomy Tube Obstruction

**DOI:** 10.7759/cureus.105561

**Published:** 2026-03-20

**Authors:** Nicole Carrillo, Yolymar Poventud, Maria Ramos-Fernandez

**Affiliations:** 1 Emergency Medicine, University of Puerto Rico, Medical Sciences Campus, San Juan, PRI

**Keywords:** acute acalculous cholecystitis (aac), gastrostomy tube, gastrostomy tube complication, geriatric emergency, rare cause of acute abdominal pain

## Abstract

Acute acalculous cholecystitis (AAC) is an inflammatory condition of the gallbladder that occurs in the absence of gallstones and is increasingly recognized among medically complex and long-term care populations. Patients receiving chronic enteral nutrition via gastrostomy tubes may be at risk for biliary complications related to impaired gallbladder emptying or mechanical factors. We describe an 81-year-old female nursing home resident with a previous medical history of hypertension, diabetes mellitus, Alzheimer’s dementia, hypothyroidism, and gastrostomy tube dependence who presented with five days of malaise, nausea, vomiting, and right upper quadrant pain. Laboratory evaluation demonstrated a cholestatic liver enzyme pattern without leukocytosis. Bedside ultrasonography revealed gallbladder dilation without gallstones. Contrast-enhanced computed tomography of the abdomen and pelvis demonstrated migration of the balloon-retained gastrostomy tube into the proximal duodenum, causing extrinsic compression of the distal common bile duct, with associated gallbladder distention and intra- and extrahepatic biliary dilatation. Magnetic resonance cholangiopancreatography confirmed obstructive physiology without choledocholithiasis. The gastrostomy tube was deflated, removed, and repositioned, resulting in rapid clinical and biochemical improvement with conservative management. In patients receiving long-term enteral nutrition, reduced physiologic gallbladder stimulation may predispose to bile stasis, creating vulnerability to obstruction when tube malposition occurs. Imaging plays a central role in diagnosis, and early recognition with prompt tube repositioning can reverse obstruction and prevent progression to more severe gallbladder injury. Clinicians should be aware that, in patients with gastrostomy tube dependence, unexplained cholestatic liver enzyme elevation should prompt assessment of tube position and evaluation for possible device-related biliary obstruction.

## Introduction

Acute acalculous cholecystitis (AAC) is an inflammation of the gallbladder that occurs in the absence of gallstones and accounts for approximately 5%-10% of all cases of acute cholecystitis [1]. Historically, AAC has been primarily described in hospitalized patients, particularly those who have experienced severe systemic stressors such as cardiac or abdominal vascular surgery, trauma, sepsis, burns, or prolonged fasting [2]. However, recognition of AAC has been increasingly reported among patients in outpatient settings [3].

Clinical manifestations of AAC may include right upper quadrant pain, nausea, vomiting, fever, leukocytosis, or abnormal liver function tests; however, no single clinical feature or laboratory abnormality reliably confirms or excludes the diagnosis [4]. Therefore, imaging is essential. Ultrasonography is typically the initial imaging modality, while computed tomography (CT) and magnetic resonance cholangiopancreatography (MRCP) are valuable when ultrasound findings are equivocal or when alternative intra-abdominal pathologies are suspected [5-7].

Beyond systemic mechanisms, AAC may also arise from mechanical or iatrogenic factors that disrupt normal biliary physiology [1-3]. Among these, gastrostomy tubes, commonly used to provide long-term enteral nutrition, represent an underrecognized source of biliary compromise. Gastrostomy tubes are widely used in patients with advanced functional dependence, which may result from neurologic impairment, cerebrovascular disease, malignancy, or other chronic illnesses [8]. Population-based data indicate that nearly 1% of hospitalized patients aged ≥65 years with dementia receive a feeding tube during hospitalization, and approximately 5%-6% of nursing home residents have gastrostomy tubes [9,10]. Although placement is generally considered safe, complications such as infection, leakage, obstruction, dislodgement, and tube migration may occur during both early and late post-procedural periods [11-12].

Gastrostomy devices may use different internal retention mechanisms. Non-balloon bumper devices are anchored by a rigid internal bolster and more commonly cause complications related to inward migration or embedding of the internal bolster within the gastric wall, or buried bumper syndrome, typically resulting from excessive tension between the internal and external bolsters [8,12]. In contrast, balloon-retained tubes remain in position through an internal water-filled balloon anchored against the gastric wall and an external fixation bumper that helps prevent inward migration [11,12]. If the external bolster loosens, the inflated balloon may advance distally through the pylorus into the duodenum [12]. Distal migration of balloon-retained tubes may cause gastric outlet obstruction and may also lead to mechanical compression of the distal common bile duct or ampulla of Vater, resulting in pancreaticobiliary obstruction [12]. While this phenomenon has been previously described in association with pancreatitis or cholangitis, to our knowledge, AAC secondary to gastrostomy tube migration has not previously been reported. Herein, we report a case of AAC caused by migration of a balloon-retained gastrostomy tube into the duodenum, resulting in mechanical biliary obstruction.

## Case presentation

An 81-year-old female nursing home resident with a medical history significant for hypertension, diabetes mellitus, Alzheimer’s dementia, hypothyroidism, and gastrostomy tube dependence for the past three months, was evaluated for a five-day history of malaise, nausea, non-bloody vomiting, and abdominal pain. Initial vital signs showed blood pressure of 109/65 mmHg, pulse rate of 79 beats/minute, respiratory rate of 18 breaths/minute, and oxygen saturation of 98%. She was afebrile at 36.8 °C. On physical examination, the patient was anicteric. She demonstrated diffuse abdominal tenderness that was most prominent in the right upper quadrant, accompanied by moderate abdominal distention. Differentials for this presentation include acute calculous and acalculous cholecystitis, ascending cholangitis, choledocholithiasis, acute pancreatitis, bowel obstruction, perforated viscus, mesenteric ischemia, and gastrostomy tube-related complications such as obstruction, dislodgement, or migration. 

**Figure 1:**
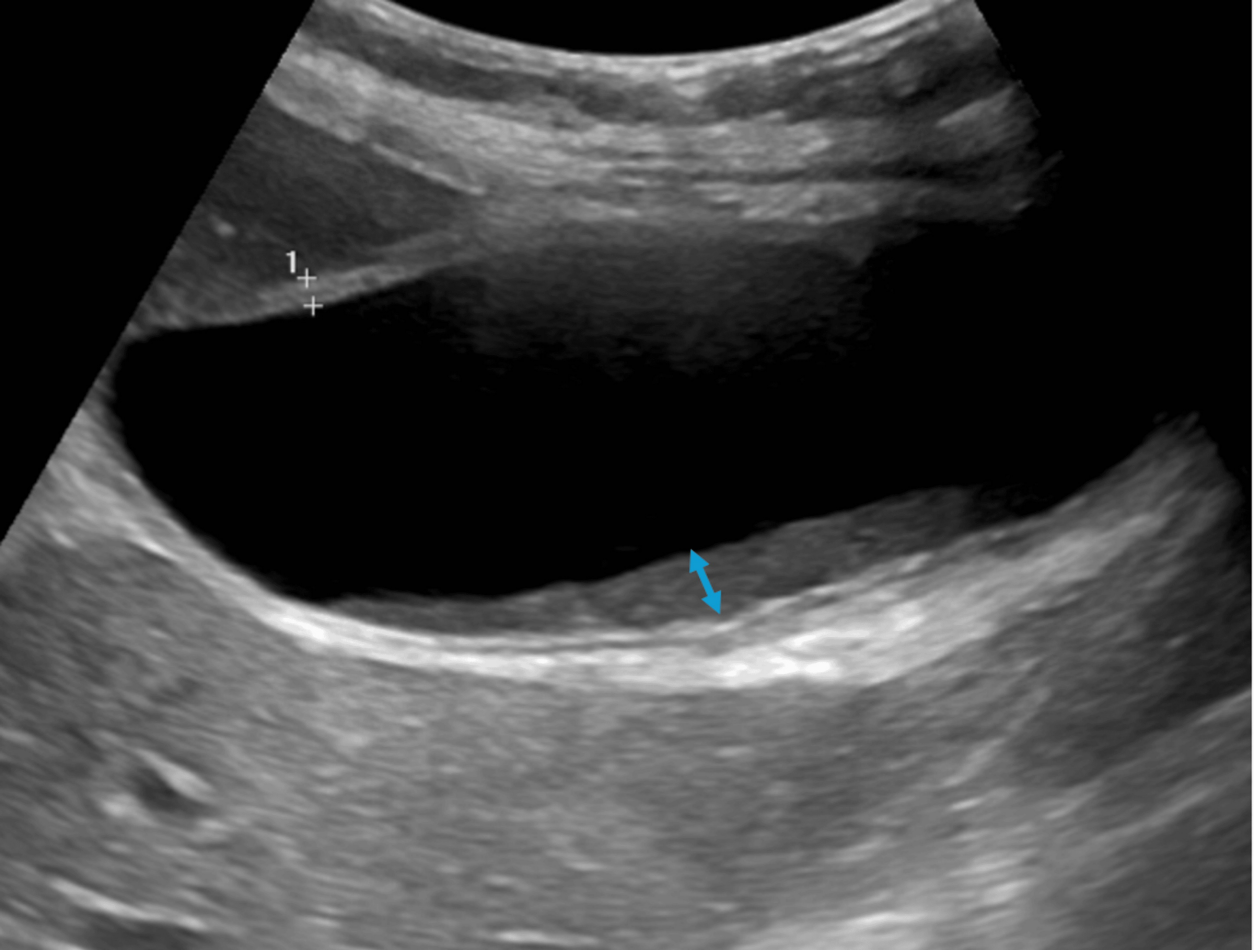
Gallbladder sludge. Right upper quadrant point-of-care ultrasonography was performed using a curvilinear transducer in the longitudinal orientation, demonstrating a distended gallbladder with a thickened wall (calipers). Dependent intraluminal echogenic material, consistent with biliary sludge (double-headed blue arrow), was also observed.

Initial evaluation included laboratory studies and bedside imaging. Laboratory testing demonstrated a hemoglobin level of 11.2 g/dL, a white blood cell count of 7.3 × 10³ cells/μL, a platelet count of 407 × 10³ cells/μL, a serum creatinine level of 0.48 mg/dL, a blood urea nitrogen level of 18 mg/dL, a glucose level of 83 mg/dL, a calcium level of 9.0 mg/dL, an albumin level of 3.4 g/dL, and a lipase level of 153 U/L. Total bilirubin was 0.84 mg/dL, and a cholestatic liver enzyme pattern was observed, characterized by elevated aspartate aminotransferase (112 U/L), alanine aminotransferase (84 U/L), and alkaline phosphatase (432 U/L). GGT was not reported. Right upper quadrant (RUQ) point-of-care ultrasonography (POCUS) was significant for maximal tenderness when the ultrasound probe was pressed over the visualized gallbladder, corresponding to a positive sonographic Murphy sign [13]. POCUS was also notable for a distended gallbladder without gallstones or wall thickening; however, biliary sludge was present (Figure 1).

**Figure 2:**
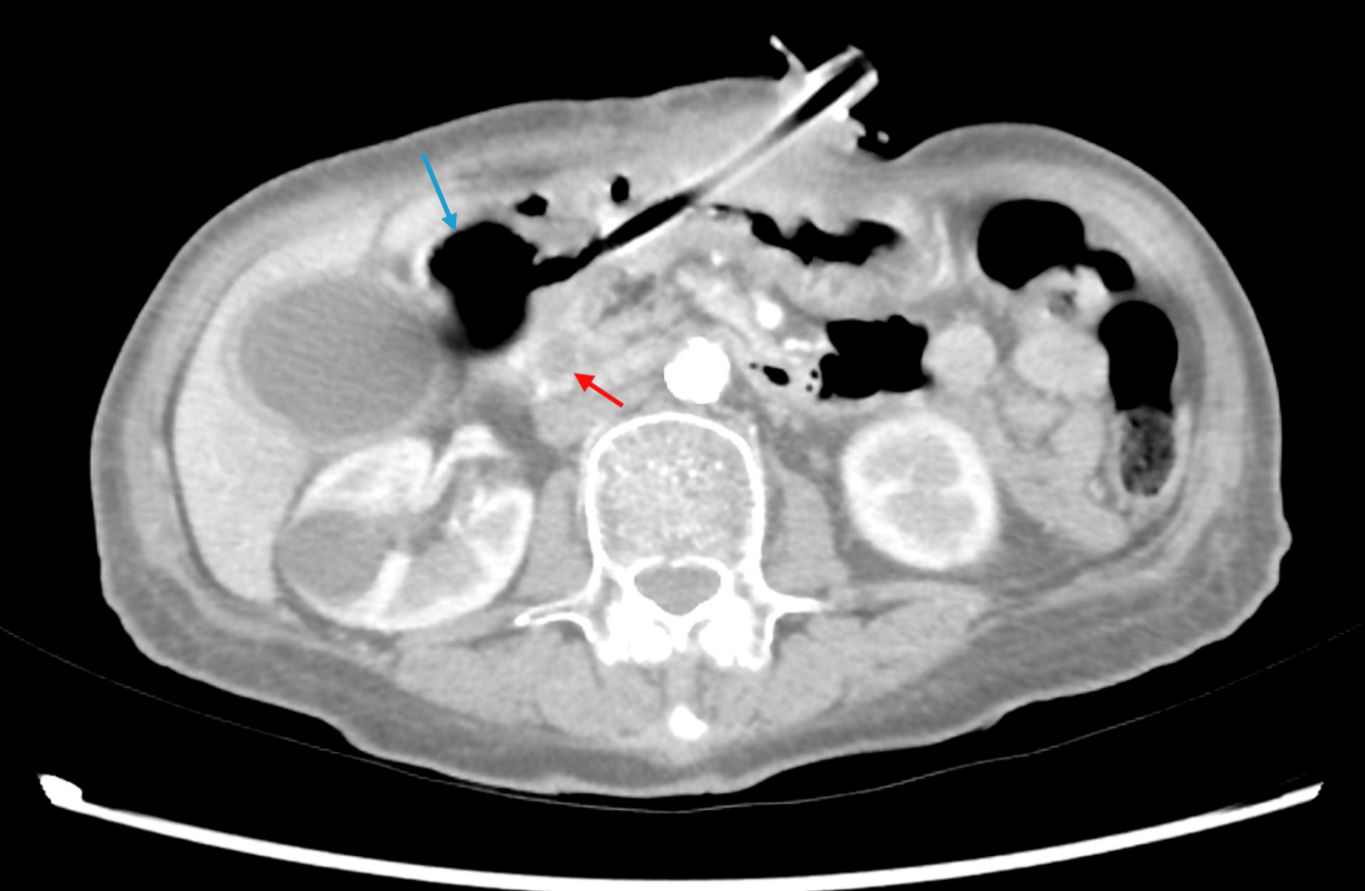
Axial computed tomography scan with intravenous contrast of the abdomen and pelvis. An axial computed tomography scan with intravenous contrast of the abdomen and pelvis demonstrated a common bile duct dilated to 11.6 mm (red arrow), secondary to mass effect from a migrated gastrostomy tube balloon in the proximal duodenum (blue arrow).

CT scan of the abdomen and pelvis with intravenous contrast demonstrated mass effect from a gastrostomy tube balloon positioned within the first portion of the duodenum, resulting in impaired biliary drainage. Associated findings included mucosal hyperenhancement of the gallbladder wall without radiodense stones, mild intrahepatic biliary ductal dilatation, and moderate common bile duct (CBD) dilatation at 11.6 mm (Figure 2).

MRCP showed a gastrostomy balloon impacted in the proximal duodenum, resulting in compression of the distal CBD, as suggested on prior CT, with dilation at 10.6 mm. MRCP also further confirmed biliary sludge with absent choledocholithiasis. The gallbladder was noted to be hydropic with wall thickening and sludge, and the pancreatic duct measured 4.1 mm. Upon consultation, the Surgery team deflated, removed, and repositioned the gastrostomy tube appropriately, resulting in relief of the mechanical obstruction. The patient was admitted for inpatient management, including bowel rest, intravenous fluids, analgesia, antiemetics, and empiric antibiotic therapy. Given marked improvement of her symptoms and ability to tolerate her feedings per gastrostomy tube, the patient was discharged in two days. 

## Discussion

The pathogenesis of AAC is multifactorial and extends beyond its traditional association with sepsis or critical illness [1]. Cholecystokinin (CCK) is a hormone that plays a central role in normal biliary physiology by stimulating gallbladder contraction and coordinating sphincter of Oddi relaxation in response to enteral nutrient exposure [14]. In patients with reduced oral intake or chronic reliance on enteral feeding - particularly among elderly and debilitated populations - CCK release may be diminished, predisposing to impaired gallbladder motility and biliary stagnation [14,15]. Prolonged bile stasis facilitates sludge formation and increases intraluminal pressure, contributing to gallbladder distention, venous congestion, and ultimately ischemic injury, which represents a recognized final common pathway in the development of AAC [1,2]. Within this physiologic context, gastrostomy tubes - although widely regarded as a safe and effective means of long-term enteral nutrition - may introduce additional mechanical and anatomic risks. Balloon-retained gastrostomy tubes rely on an intragastric inflatable balloon to maintain luminal positioning, while external stability is provided by an external bolster [8]. Inadequate external fixation allows inward migration of the tube along the mature gastrostomy tract [12]. In addition, gastric peristalsis generates repetitive anterograde forces that may progressively advance the compliant balloon across the pylorus, particularly during periods of increased gastric motility [15]. Other contributing factors include patient repositioning, inadvertent tube manipulation during routine care, and prolonged supine positioning [16]. Distal migration of balloon-retained gastrostomy tubes has been associated with rare mechanical complications involving adjacent gastrointestinal and periampullary structures [17]. Although acute acalculous cholecystitis has not previously been described in this context, the proposed mechanism in this case is comparable to other obstructive complications reported in the literature (Table 1).

**Table 1:** Mechanical complications of gastrostomy tube balloon migration and their proposed mechanisms.

Complication	Mechanism
Gastric outlet obstruction	Distal migration of a balloon gastrostomy tube through the pylorus allows the inflated balloon to lodge within the duodenal bulb or proximal duodenum, producing obstruction of gastric emptying and gastric outlet obstruction [17-19].
Acute pancreatitis	Migration of the balloon into the second portion of the duodenum may compress the periampullary region or obstruct the ampulla of Vater, impairing pancreatic outflow and triggering acute obstructive pancreatitis [20-23].
Ulceration and gastrointestinal bleeding	Persistent balloon impaction against the pyloric channel or duodenal mucosa may produce localized ischemia and pressure necrosis [24]. Progressive mucosal injury can result in ulcer formation and gastrointestinal bleeding [24,25].
Duodenal perforation	Prolonged distal migration of a balloon gastrostomy tube into the duodenum may result in sustained pressure on the duodenal wall, leading to ischemia, pressure necrosis, and deep mucosal ulceration that may progress to perforation in severe cases [26,27].
Retrograde intussusception	A migrated balloon gastrostomy tube within the proximal small bowel may act as a lead point for bowel telescoping, permitting retrograde jejunogastric or duodenojejunal intussusception [28,29].
Small bowel obstruction	Continued distal migration beyond the proximal duodenum may allow the inflated balloon to become impacted within the small bowel lumen, functioning as a mechanical transition point and producing small bowel obstruction [18,30].
Gastrocolocutaneous gastrocolic fistula	Gastrocolocutaneous fistulas may occur when the gastrostomy tract traverses the colon during initial placement or when chronic pressure from the internal balloon leads to erosion of the gastric or colonic wall, creating a tract between the stomach, colon, and skin [31-33].
Gastric volvulus	Distal migration of a balloon gastrostomy tube into the duodenal bulb may tether the gastric outlet and act as a pivot point for gastric rotation, allowing the stomach to twist along its axis and producing gastric volvulus [34-36].
Acute acalculous cholecystitis	Distal migration of a balloon gastrostomy tube into the second portion of the duodenum may compress the periampullary region and impair biliary drainage [20,21]. Resulting bile stasis and gallbladder distention may lead to inflammatory injury of the gallbladder wall and acute acalculous cholecystitis [1,37,38].

In the present case, the balloon had migrated across the pylorus and was lodged within the first portion of the duodenum, the superior segment known as the duodenal bulb. This segment lies immediately proximal to the descending second portion of the duodenum, which contains the major duodenal papilla, where the distal common bile duct and pancreatic duct empty into the intestinal lumen through the ampulla of Vater [39]. Balloon impaction within this region may therefore produce mechanical compression of the periampullary area and impair biliary drainage [5]. When the gastrostomy balloon becomes impacted in this segment, it can exert focal extrinsic compression on the periampullary region or distal common bile duct, impairing bile flow and producing functional biliary obstruction [40]. Such obstruction results in progressive upstream biliary dilatation that may be visualized on cross-sectional imaging modalities such as CT or MRCP [41].

In our patient, the CT and MRCP findings of proximal biliary dilatation can therefore be anatomically explained by balloon impaction within the proximal duodenum, causing compression of the ampullary complex and subsequent impaired biliary drainage [40]. Similar reports have demonstrated rapid clinical and biochemical improvement following balloon deflation and repositioning or removal of the gastrostomy tube, further supporting the mechanical and reversible nature of this process [23,40,41]. Preventive strategies described in the literature include careful positioning of the external bolster approximately 1-2 cm from the skin to avoid excessive tension, documentation of the external tube length at the skin level at the time of placement, and routine verification of these measurements during nursing care to detect early inward migration [17,40].

In older adults receiving long-term enteral nutrition, AAC and other biliary pathologies often present with nonspecific symptoms, complicating early recognition [2]. While feeding intolerance or abdominal distention may occur, early deterioration often manifests as functional or neurocognitive changes, before overt abdominal findings emerge [9,10]. When laboratory evaluation reveals an otherwise unexplained cholestatic pattern, further assessment for impaired biliary drainage should be pursued, particularly when a potential mechanical source of obstruction is present. In this setting, imaging plays a critical role in establishing the diagnosis. Diagnostic frameworks, including the Tokyo Guidelines 2018, support the diagnosis of acute cholecystitis based on a combination of clinical, laboratory, and imaging findings [4,5]. Characteristic imaging features may include gallbladder wall thickening ≥3-4 mm, gallbladder distention, pericholecystic fluid, subserosal edema, intramural gas, mucosal sloughing, biliary sludge, or gallbladder hydrops [4-6]. In the present case, cross-sectional imaging demonstrated gallbladder distention, wall thickening, and biliary sludge, findings compatible with these diagnostic imaging features [4,5]. Although systemic inflammatory markers were absent, atypical presentations without leukocytosis or fever are well recognized among elderly and debilitated patients with acute acalculous cholecystitis [2].

Ultrasonography is the first-line modality for AAC, given its availability and its ability to identify gallbladder distention, sludge, and bile duct dilatation; however, its sensitivity ranges from approximately 50% to 70% [4]. When ultrasonography is nondiagnostic or a mechanical etiology is suspected, contrast-enhanced CT offers higher diagnostic performance, with reported sensitivity of approximately 90%, and is better suited to detect complications and identify extrinsic causes of biliary obstruction, including device malposition or duodenal compression [4]. MRCP provides high-resolution, noninvasive visualization of the pancreaticobiliary ductal system and demonstrates sensitivity of approximately 90%-95% for detecting choledocholithiasis, making it useful for excluding obstructive biliary pathology [4,7].

## Conclusions

Clinicians should maintain a high index of suspicion for AAC in patients with gastrostomy tube dependence who develop otherwise unexplained clinical deterioration. Although AAC is classically associated with severe systemic stressors, mechanical and iatrogenic factors such as gastrostomy tube migration remain underrecognized causes of biliary obstruction. In patients presenting with unexplained cholestatic liver enzyme abnormalities, acute abdominal pain, or systemic decline, clinicians should carefully inspect the gastrostomy tube, verify the external bumper position and tube length at the skin level, and obtain appropriate imaging to evaluate for possible tube migration before pursuing invasive diagnostic procedures.

In this case, early recognition and prompt deflation, removal, and repositioning of the migrated gastrostomy tube, a bedside intervention that can often be safely performed in the emergency department by trained clinicians, resulted in rapid clinical and biochemical improvement while avoiding more invasive interventions. Preventive strategies are therefore essential and must include proper positioning of the external bolster to prevent inward migration, documentation of the tube length at the time of placement, and routine assessment of the stoma site and tube length during daily nursing care. Education of caregivers and healthcare personnel regarding signs of tube malfunction, such as feeding intolerance, abdominal distention, leakage, or unexplained laboratory abnormalities, may facilitate earlier recognition of tube migration. Therefore, incorporating structured gastrostomy care practices and multidisciplinary follow-up may further reduce device-related complications and improve outcomes in patients requiring long-term enteral nutrition. 
